# A Novel Approach for 3D-Structural Identification through Video Recording: Magnified Tracking †

**DOI:** 10.3390/s19051229

**Published:** 2019-03-11

**Authors:** Yunus Emre Harmanci, Utku Gülan, Markus Holzner, Eleni Chatzi

**Affiliations:** 1 Institute of Structural Engineering, Department of Civil, Environmental and Geomatic Engineering, ETH Zürich, 8093 Zurich, Switzerland; harmanci@ibk.baug.ethz.ch; 2 Institute of Environmental Engineering, Department of Civil, Environmental and Geomatic Engineering, ETH Zürich, 8093 Zurich, Switzerland; utku.guelan@ifu.baug.ethz.ch (U.G.); holzner@ifu.baug.ethz.ch (M.H.)

**Keywords:** vibration-based measurement, SHM, structural identification, motion magnification, particle tracking velocimetry

## Abstract

Advancements in optical imaging devices and computer vision algorithms allow the exploration of novel diagnostic techniques for use within engineering systems. A recent field of application lies in the adoption of such devices for non-contact vibrational response recordings of structures, allowing high spatial density measurements without the burden of heavy cabling associated with conventional technologies. This, however, is not a straightforward task due to the typically low-amplitude displacement response of structures under ambient operational conditions. A novel framework, namely *Magnified Tracking* (MT), is proposed herein to overcome this limitation through the synergistic use of two computer vision techniques. The recently proposed phase-based motion magnification (PBMM) framework, for amplifying motion in a video within a defined frequency band, is coupled with motion tracking by means of particle tracking velocimetry (PTV). An experimental campaign was conducted to validate a proof-of-concept, where the dynamic response of a shear frame was measured both by conventional sensors as well as a video camera setup, and cross-compared to prove the feasibility of the proposed non-contact approach. The methodology was explored both in 2D and 3D configurations, with PTV revealing a powerful tool for the measurement of perceptible motion. When MT is utilized for tracking “imperceptible” structural responses (i.e., below PTV sensitivity), via the use of PBMM around the resonant frequencies of the structure, the amplified motion reveals the operational deflection shapes, which are otherwise intractable. The modal results extracted from the magnified videos, using PTV, demonstrate MT to be a viable non-contact alternative for 3D modal identification with the benefit of a spatially dense measurement grid.

## 1. Introduction

System identification [1,2,3] and structural health monitoring [4,5] methods are increasingly gaining industry acceptance as a complementary tool in the life-cycle assessment of engineered systems. Currently, the most common choice for structural instrumentation lies in the use of wired accelerometers; this is due to the wide availability of mid-level precision sensors at a fairly low cost. Such sensors may be permanently installed on a structure at discrete locations; a task which is not always straightforward because of accessibility issues, the heavy cabling work involved, as well as the increased costs of data acquisition [6]. Lowering the spatial density as a means of compensating may bear the consequence of sacrificing accuracy in system identification and damage detection. Another alternative is offered via wireless sensing technologies [7,8], albeit at the cost of a short battery life and limited transmission potential for dynamic measurements [9,10].

On the other hand, non-contact image-based techniques have recently offered an alternative for low-cost dynamic measurements and structural health monitoring applications [11]. As regards the outside use of dynamic displacement measurements, applications can be found in crack detection with the intention of enhancing visual inspection capabilities by employing techniques, such as object detection and grouping [12], multi-sequential image filtering [13], or region-based deep learning [14]. When it comes to obtaining dynamic displacements, different approaches have been proposed based on template matching (digital image correlation) [15,16], feature point matching [17], and optical flow [18,19]. The latter technique received particular interest with the emergence of a video manipulation technique, namely the phase-based motion magnification (PBMM) [20]. PBMM relies on revealing imperceptible motion in videos via the utilization of complex-valued steerable pyramids within defined frequency bands.

The potential of PBMM for inferring modal motion has been explored in numerous studies. Chen et al. [21] extracted mode shapes of a simple cantilever structure using PBMM and optical flow and compared these with laser vibrometer measurements. This was later extended by Cha et al. [22] to detect damage on similar cantilever beams through the use of an unscented Kalman filter. Yang et al. [23] implemented a modified version of PBMM that can blindly extract modal frequencies, damping ratios, and continuous mode shapes as an output-only method. This was achieved via principal component analysis for dimensionality reduction and subsequent complexity pursuit for blind mode identification. Further extensions of this method includes a technique to obtain modal information from videos with frame-rates lower than the Nyquist frequency [24], as well as non-ideal operating conditions [25]. The principle was further adopted in the context of damage detection for cantilever structures using spatial fractal dimension analysis [26]. Sarrafi et al. [27] performed tests on healthy and damaged wind turbine blades and estimated their operational deflection shapes via PBMM and phase-based motion estimations. The use of this concept in outdoor settings was recently demonstrated by Shang and Shen [28] on steel bridges, additionally employing a maximum likelihood estimation scheme to automize the selection of frequency bands to be magnified. Fioriti et al. [29] utilized PBMM for modal identification of a full-scale large historic masonry structure, the Ponte delle Torri in Spoleto, by using videos taken from a common smartphone device. It should be noted that as large structures are usually characterized by fundamental frequencies lower than 5 Hz, the use of such low-sampling devices might prove promising. Interested readers are referred to Xu and Brownjohn (2017) [30] for a review of the computer-vision techniques for structural health monitoring (SHM) applications.

The aforementioned works mainly focus on two-dimensional techniques, which might be a limiting factor for some structures either because of the constraints in camera placement or to the out-of-plane motion (with respect to the camera) of the structure itself. This issue was addressed by various researchers utilizing a variety of techniques for cases with perceptible structural response. To name a few, Park et al. [31] obtained dynamic 3D displacements of a shear frame via a motion capture system (MCS). Baqersad et al. [32] and Patil et al. [33] utilized 3D digital image correlation (DIC) to obtain dynamic displacements and operational mode shapes of structures with complex geometries. Wang et al. [34] identified the model parameters of a car bonnet by extracting the shape features from 3D-DIC measurements employing an adaptive geometric moment descriptor. Recently, the out-of-plane vibrations of cantilever beams were tracked via a new camera technology, namely light field imagers, by Chesebrough et al. [35]. To the best of the authors’ knowledge, studies in three-dimensions with imperceptible motion employing PBMM are currently limited to the use of the 3D-DIC technique [36,37]. Particle tracking velocimetry (PTV) was proposed by the authors as an alternative technique to obtain dynamic displacements for SHM applications, in both 2D [38] and 3D [39,40], as well as a performance comparison with optical flow [19]. PTV, which is an open source optical measurement technique [41], has been in use for decades in diverse engineering applications from the macro to micro scale. Applications include, but are not limited to, assessing the vorticity dynamics [42] and the dissipation of kinetic energy in turbulent flows [43], investigating the blood flow patterns for different cardiovascular diseases [44,45], tracking bird and fish migrations [46,47], the validation of computational fluid dynamics simulations [48,49], and the validation of magnetic resonance imaging (MRI)-based flow measurements [50]. However, PTV has so far been limited to assessing high-amplitude displacements, which does not represent the amplitude range of many civil engineering structures.

In this study, we propose a framework for vision-based structural identification via the synergistic use of two computer vision techniques. Magnified tracking (MT) first employs phase-based motion magnification to reveal modal information from videos with imperceptible motion and then PTV to track amplified motion of small markers placed on the structure. A novelty of this work lies in the use of the proposed framework both in 2D as well as 3D with high temporal and spatial density. The capabilities of MT for all range of motions, and the limitations of PTV for the so-called perceptible motion range are demonstrated by comparing results of a lab-scale shear-frame that is deployed with conventional sensors.

## 2. Materials and Methods

### 2.1. Methodology

In this study, we propose a novel image-based method, MT, which fused PTV and PBMM and we further compare the proposed method against conventional displacement measurement techniques, e.g., accelerometer, laser and linear variable differential transformer (LVDT) measurements, and computational simulations.

#### 2.1.1. Particle Tracking Velocimetry

PTV is a standard method for the time-resolved measurement of the displacement of markers (e.g., tracer particles in fluid flow or markers on moving objects) in a three-dimensional space. The main principle lies in the video recording (using a multi-camera setup) of moving markers, illuminated with a light source, for subsequent stereo reconstruction of 3D marker positions and their tracking in time. Spatial coordinates of particles/markers are computed by optical triangulation utilizing the collinearity condition of the projective geometry of imaging [51]. The formulation of the collinearity equation, which contains three coordinates $X_{0}$, $Y_{0}$, $Z_{0}$ of the projective center and three rotation angles describing the direction of the optical axis, may be formulated as provided in Equations (1)–(3) [41]:

(1) $$\left[\begin{array}{c} x_{i}-x_{h}\\ y_{i}-y_{h}\\ z_{i}-z_{h} \end{array}\right]=\lambda_{i}\cdot R\cdot \left[\begin{array}{c} X_{i}-X_{0}\\ Y_{i}-Y_{0}\\ Z_{i}-Z_{0} \end{array}\right]$$

(2) $$x_{i}=x_{h}-c\cdot \frac{r_{11}\left(X_{i}-X_{0}\right)+r_{21}\left(Y_{i}-Y_{0}\right)+r_{31}\left(Z_{i}-Z_{0}\right)}{r_{13}\left(X_{i}-X_{0}\right)+r23\left(Y_{i}-Y_{0}\right)+r_{33}\left(Z_{i}-Z_{0}\right)}$$

(3) $$y_{i}=y_{h}-c\cdot \frac{r_{12}\left(X_{i}-X_{0}\right)+r_{22}\left(Y_{i}-Y_{0}\right)+r_{32}\left(Z_{i}-Z_{0}\right)}{r_{13}\left(X_{i}-X_{0}\right)+r23\left(Y_{i}-Y_{0}\right)+r_{33}\left(Z_{i}-Z_{0}\right)},$$

where $x_{i}$, $y_{i}$, and $z_{i}$ are the image coordinates, $x_{h}$, $y_{h}$, and $z_{h}$ are image principle points (camera parameter), *c* is the image principle distance (camera parameter), $\lambda_{i}$ is the scale factor, *R* is the 3 × 3 rotation matrix, $r_{ij}$ are the elements of the rotation matrix, $X_{i}$, $Y_{i}$, $Z_{i}$ are object point coordinates, and $X_{0}$, $Y_{0}$, $Z_{0}$ are the camera projective center coordinates.

Two camera views are enough for stereoscopic matching. In our experiments, we use four camera views to enhance the robustness and accuracy of the approach. The stereo reconstruction requires an appropriate calibration of the cameras. The calibration, which can be either dynamic (with a moving calibration target) or static (with images of calibration targets), determines the external and internal camera parameters, e.g., position, orientation, and focal length [52]. In our measurements, a static calibration target was used with a regular array of 20 × 30 points with a grid distance of 56 mm.

The tracking of a particle is predicted under the assumption of constant velocity $u_{p}$, using a three-dimensional particle position $x_{p}$ at time step *t*, and a position $x_{p}\left(t+\Delta t\right)$ at the consecutive time step t+Δt, as derived in Equation (4). Subsequently, linking of the particle positions at time steps *t* and t+Δt can be realized via linear extrapolation [53]. It should be noted that the time increment Δt corresponds to the frame rate of the recording.

(4) $$x_{p}\left(t+\Delta t\right)=x_{p}\left(t\right)+u_{p}\cdot \Delta t$$

**Figure 1:**
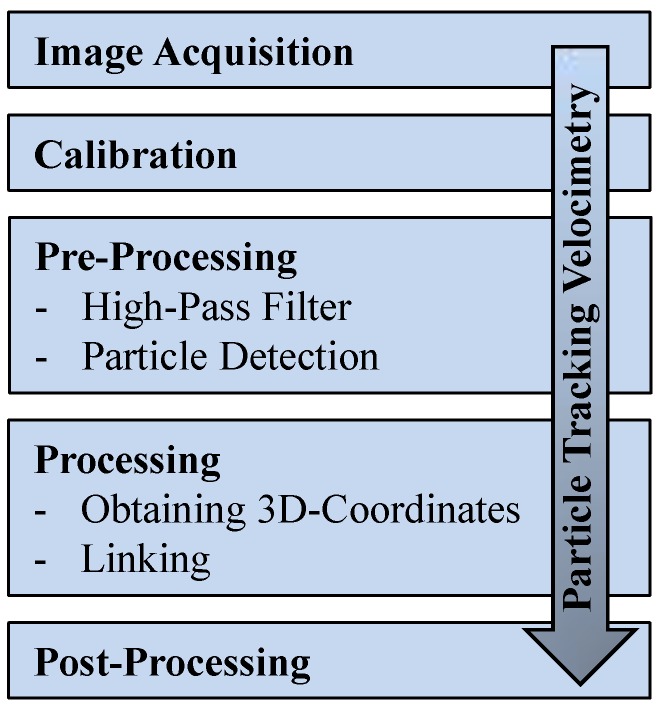
Workflow process of the 3D particle tracking velocimetry (PTV) algorithm to assess the displacement of tracer markers.

As described in Figure 1, an initial pre-processing step is required after image acquisition and calibration. First, images are high-pass filtered to remove pixel noise. Image coordinates of all markers are detected relying on grey value intensity and connectivity on the 2D image. The center of the detected markers in the image domain is calculated as the arithmetic mean of the pixel coordinates weighted by the associated grey value intensity. Stereoscopic matching of markers is realized by means of calibrated camera orientation parameters and the position information extracted from all four stereoscopic camera views. This is achieved via epipolar line intersection to extract the 3D positions of each marker [54]. Linking of the 3D positions of individual markers in time results in establishing Lagrangian trajectories, which allow calculating displacements.

#### 2.1.2. Phase-Based Motion Magnification

Phase-based motion magnification is a computer vision technique that allows one to manipulate motions in videos. It relies on the modification of local phase variations of the complex-valued steerable pyramid coefficients over time in different spatial scales and orientations. By adopting sub-octave bandwidth complex steerable pyramids to decompose each frame in different scales and orientations, their amplitude and phase can be separated. The phase information at every location, orientation, and scale is then temporally filtered, amplified, and then reconstructed into a video [20]. The complex steerable pyramids can be imagined to operate in a similar fashion to a spatially localized Fourier transform [55].

In its simplest form, let us assume a 1D intensity signal representing translation within an image. Assuming an intensity profile f(x+δ(t)) where δ(t) corresponds to displacements, an α-times magnified motion f(x+(1+α)δ(t)) can be achieved by utilizing a global Fourier basis. In the case of videos, where motion is not global and so the local displacements are instead in the form δ(x,t), the complex steerable pyramids are utilized to cope with local motion. For the 1D example, the original intensity profile can be rewritten as a sum of complex sinusoids as shown in Equation (5).

(5) $$f\left(x+\delta \left(t\right)\right)=\Sigma_{\omega =-\infty }^{\infty }A_{\omega }e^{i\omega (x+\delta (x))}$$

The band at a specific frequency ω can be written as

(6) $$S_{\omega }\left(x,t\right)=A_{\omega }e^{i\omega (x+\delta (t))},$$

where its phase (ω(x+δ(t))) encompasses information about the motion.

**Figure 2:**
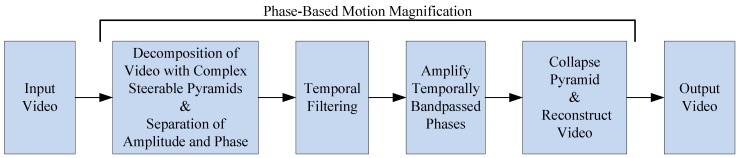
Workflow process of the phase-based motion magnification (PBMM) algorithm to amplify motion in a video within a defined frequency band.

The phase is then temporally filtered via a DC bandpass filter in order to remove the ωx component. Subsequently, the filtered phase can be multiplied by a factor of α to obtain a magnified motion at sub-band ω, as demonstrated in Equation (7). Finally, a flowchart of the whole process is provided in Figure 2.

(7) $${\hat{S}}_{\omega }\left(x,t\right)=A_{\omega }e^{i\omega (x+(1+\alpha )\delta (t))}.$$

More information on the above-given equations and its framework is provided by Wadhwa et al. [20] and Rubinstein [56]. Although not explored within this work, the cited documents further demonstrate that PBMM can also be used to attenuate motion, which can be used within this context to either: (i) filter out unwanted motion due to cyclic events; or (ii) remove atmospheric turbulence in long-distance measurement situations. The use of this particular technique within the structural identification domain offers significant potential as a result of the videos of structures magnified around the structure’s natural frequencies revealing their respective operational deflection shapes.

#### 2.1.3. Magnified Tracking

Magnified tracking is a video recording-based measurement framework proposed by the authors, which utilizes both aforementioned algorithms to extract modal information with high-spatial density from a large to imperceptible motion range in 2D and 3D. This implies that it can be applied across diverse conditions, i.e., from experimental/forced testing to operational regimes.

The framework relies on the use of markers placed on the structure of interest, which are subsequently tracked via the PTV algorithm. When dealing with sufficiently large (perceivable) motion, PTV can be directly utilized to extract a dynamic displacement response of the recorded structure. When dealing with imperceptibly small (subtle) motions, first PBMM is employed to amplify the motion within a certain frequency band, which generally corresponds to the natural frequencies of the structure that can be reliably obtained through a single conventional (e.g., MEMs-based) sensor. The amplified motion reveals operational deflection shapes at the frequency of interest [57], and its Lagrangian trajectories can be tracked at discrete marker locations via PTV. Snapshots from this tracked motion correspond to the operational deflection shapes and do not require further processing.

**Figure 3:**
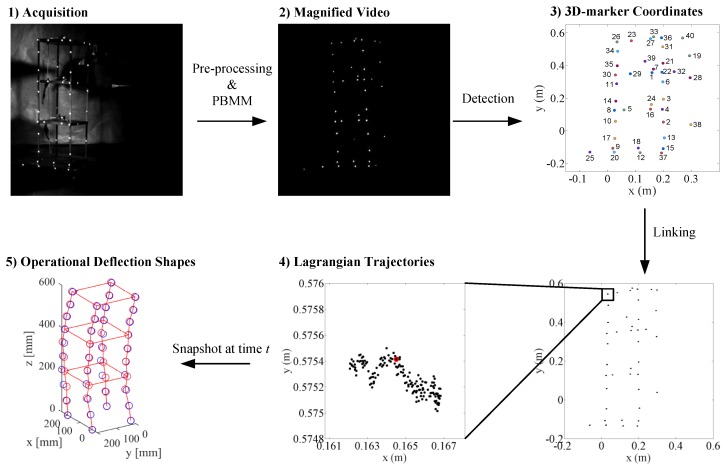
Processing steps of magnified tracking (MT) including pre-processing and PBMM, detection, linking, and modal analysis.

The processing steps of 3D-MT are presented in Figure 3, which offers an example for the case of subtle motion. For the perceptible motion range, PBMM is not necessary after pre-processing, since PTV can, in such a case, successfully track the displacements on its own.

### 2.2. Experimental Investigation

**Figure 4:**
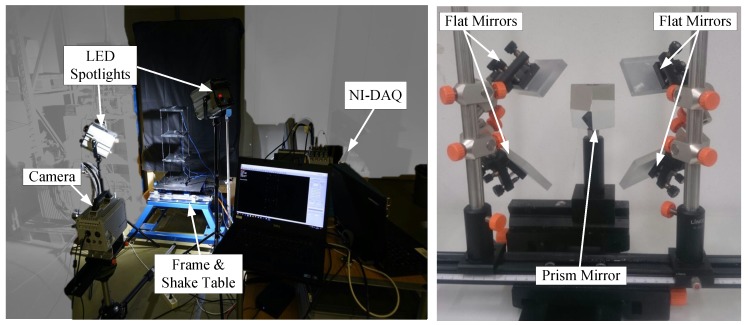
View of the experimental setup including the frame, shake table for triggering the motion, camera for recording the motion of the markers on the structure, LED spotlights for illuminating the investigation domain (**left**) and optical setup including image splitter and mirrors (**right**).

An experimental campaign was launched for a comparative assessment of the proposed measurement framework against conventional sensors. Two separate experimental setups were prepared, one for the 2D and one for the 3D camera configuration. For this purpose, a three-storey shear frame with an idealized storey height of 200 mm and a floor area of 200 × 200 mm${}^{2}$ was constructed. Columns were made of steel with a cross section of 10 × 3 mm${}^{2}$. Plates were made of aluminum with a thickness of 15 mm. White paper sticker markers with a diameter of 2 mm were introduced onto the structure on each storey as well as at every third of each column. A uniaxial shaking table (Quanser, Markham, ON, Canada) was used in both experimental setups to induce “large-scale” (perceptible) motion through either sinusoidal or earthquake ground excitation (1994 Northridge). The operational bandwidth of the shake table was limited to 20 Hz, hence no random ground motion was implemented as part of this testing campaign. An impact hammer (Kistler, Winterthur, Switzerland) was used for impact testing, inducing a visually imperceptible structural response. All excitations presented in this work are listed in Table 1. All sensors were connected to a 16 channel NI-DAQ data acquisition system (National Instruments, Austin, TX, USA). The frame was illuminated via two high-power single LED spotlights (HEDLER Profilux, Runkel, Germany) placed diagonal to the frame to enhance contrast. Figure 4 presents the key-components of the experimental setup. Both the 2D and 3D cases feature conventional sensors in addition to the camera configuration.

Measurements for the proposed framework were obtained by following a fairly straightforward testing protocol. A Fastcam SA5 high-speed camera (Photron, Tokyo, Japan) was employed for recording instantaneous motion, which allows one to record 1.56 s at a full resolution of 1024×1024 pixels and a maximum frequency of 7000 fps with 16 GB memory. A 12-bit analog digital converter (ADC) (Bayer system color, single sensor) with 20μm pixel sensor provided higher light sensitivity for high-speed recordings. A Nikon AF Micro-Nikkor 28 mm f/2.8D lens was used for these experiments.

Contrary to conventional multi-camera configurations, an image splitter system was used to mimic four different views by using a single camera. The four-way splitter consisted of a set of four fixed primary slanted mirrors assembled on a regular pyramid and four secondary mirrors [58], which were arranged such that the acquired images for all four mirrors covered the same domain, within the focused distance set by the lens [52]. In this study, the camera was positioned at a distance of 2 m from the frame, with approximately a $45^{\circ }$ horizontal angle with respect to the object.

#### 2.2.1. 2D Experimental Setup

**Figure 5:**
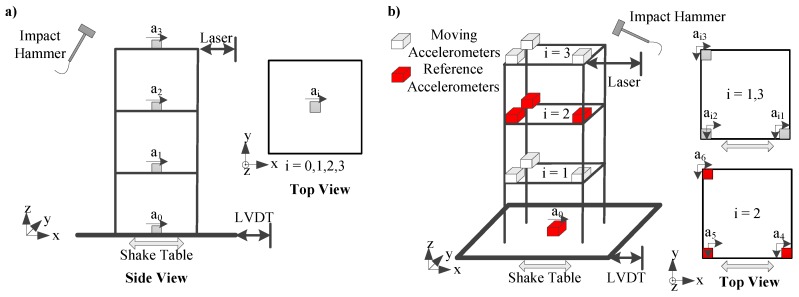
Test setup and sensor layout for: (**a**) 2D; and (**b**) 3D measurements.

For the 2D setup, four piezoelectric uniaxial accelerometers (PCB Piezotronics, Depew, NY, USA) were used, all placed at the centroid of each storey. Displacements were measured via an LVDT (HBM GmbH, Germany) at the shake-table base and by a laser displacement sensor (SICK, Waldkirch, Germany) directed towards a corner of the third storey. The test setup and sensor layout of the 2D configuration is presented in Figure 5a. Measured excitation scenarios are shown in Table 1. For the 2D-PTV measurements, the spatial resolution of the images was 1024 × 1024 pixels and the temporal resolution was 500 fps.

#### 2.2.2. 3D Experimental Setup

For the 3D setup a uniaxial accelerometer was used at the base and six piezoelectric triaxial accelerometers (PCB Piezotronics, Depew, NY, USA) were used at the upper floors. As a result of the insignificant structural response along the *z*-axis, accelerations were measured only on the *x*- and *y*-axes. In order to capture torsional modes, three accelerometers were installed per storey. However, because of the channel limitations, it was not possible to employ all storeys at the same time. For this purpose, a reference-based experimental modal analysis procedure was adopted [59,60]. Three accelerometers on the second floor (and the base sensor) were kept fixed in every experiment and the remaining three were placed either at the first or third storey. Both the shake table and the frame was rotated approximately $30^{\circ }$–$45^{\circ }$ in order to have out of plane displacement components with respect to the camera. The spatial resolution of the images in the 3D-PTV measurements was 512 × 512 pixels as a consequence of the image splitter and the temporal resolution was 500 fps. The test setup and the sensor layout are presented in Figure 5b.

### 2.3. Numerical Modelling

A numerical model of the shear frame was constructed in SAP2000 [61] in order to collaborate the experimental measurements, especially at locations where markers were placed for tracking but no conventional sensors were installed. The model, comprising beam elements for columns and shell elements for slabs, was updated and validated relying on the use of accelerometer measurements.

## 3. Results and Discussion

A series of measurements were performed for different types of excitations, as shown in Table 1. The comparison between the proposed methods and various reference sensors, e.g., LVDT, laser and accelerometers, were made both in the time and frequency domains.

### 3.1. 2D-PTV

**Figure 6:**
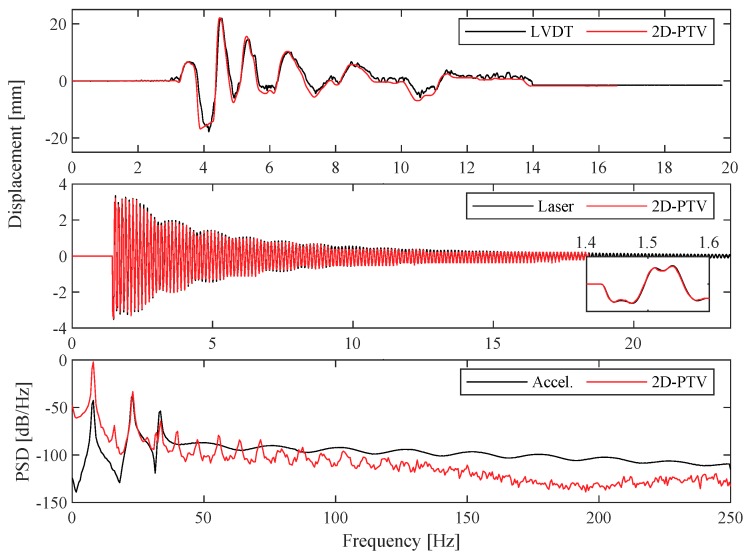
(**Top**) Displacement validation of 2D-PTV against LVDT for Northridge input at shake-table base. (**Middle**) Displacement validation of 2D-PTV against laser distometer for a hammer impact excitation at the top of the frame. (**Bottom**) Power spectral density validation of 2D-PTV against an accelerometer for a hammer impact excitation at the top of the frame.

2D-PTV measurements were performed for two different base excitations, as shown in Table 1. The displacements obtained through 2D-PTV were compared against LVDT measurements for the Northridge earthquake excitation at the shake-table base and the laser at the top of the structure for a hammer impact test, both of which are plotted in Figure 6. A good match is obtained at both points, and displacements obtained at the table base contained less noise compared to the LVDT measurements. Power spectral densities (PSD) estimated from the accelerometer and its corresponding marker at the top floor following a hammer impact are compared against and provided in Figure 6. PSD estimates were computed using a Hamming window with 1024 samples and 50% overlap, and the displacement/acceleration signals were only de-meaned and not filtered for comparison purposes. Initial observation indicates a close correlation between the two measurement techniques, with the potential existence of harmonic frequency peaks of the 2D-PTV results.

**Figure 7:**
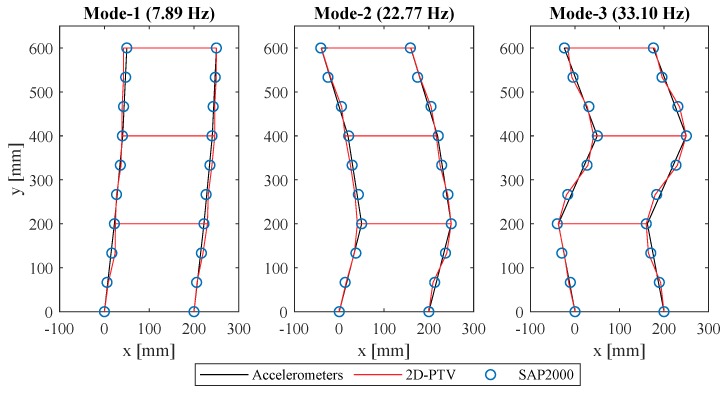
First three mode shapes obtained via accelerometers, 2D-PTV, and SAP2000.

Further validation of the proposed framework was carried out via modal analysis. The Eigensystem realization algorithm (ERA) was employed to analyze the structural response following hammer impacts. The stabilization diagrams, and corresponding identified frequencies, reveal that the 2D-PTV measurements closely correlate to the accelerometer measurements in the frequency domain. The identified natural frequencies are given in Table 2. Mode shapes obtained through the accelerometers, the 2D-PTV displacements, and the SAP2000 model are compared against each other, as presented in Figure 7. Information about the third mode was not captured by 2D-PTV since it corresponds to a torsional mode shape. The SAP2000 model is utilized to provide a reference with higher spatial density to compare PTV measurements against. Both measurement techniques correlate very well to the SAP2000 model at the floor levels; however, the accelerometers do not provide information along the columns, which are nonetheless adequately measured by the 2D-PTV via markers at those locations. The accuracy of displacements obtained from 2D-PTV is further quantitatively validated by means of the cross modal assurance criterion (cross-MAC) values, provided in Table 3. 2D-PTV provides cross-MAC values very close to one, both against the accelerometer-based, as well as the SAP2000 results.

### 3.2. 3D-PTV

**Figure 8:**
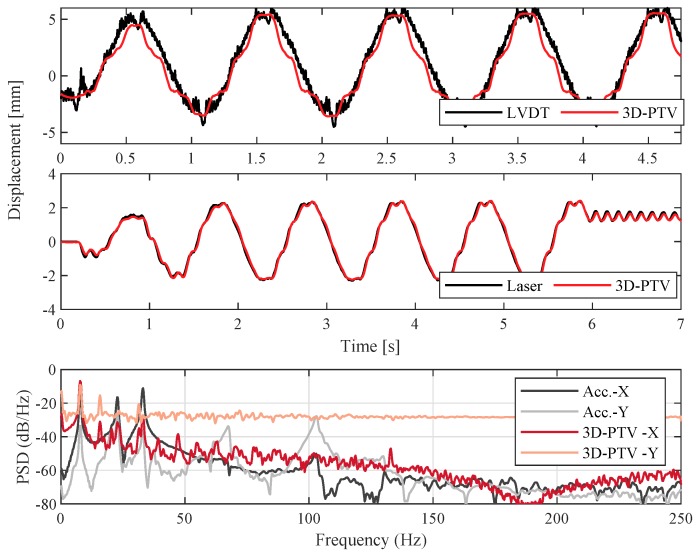
(**Top**) Displacement validation of 3D-PTV against LVDT for a sinusoidal input at shake-table base. (**Middle**) Displacement validation of 3D-PTV against laser distometer for a sinusoidal input at top of the frame. (**Bottom**) Power spectral density validation of 3D-PTV against an accelerometer for a hammer impact excitation at top of the frame.

As the camera was positioned at a $45^{\circ }$ angle with respect to the shake table and frame assembly in the measurement scenarios involving the 3D camera setup, the obtained displacements contained both in-plane (*x*-) and out-of-plane (*y*-) components. These components were converted to the shake table’s axis in order to compare against the LVDT and laser measurements. Displacements obtained via LVDT at shake-table base and laser at the top of the frame are compared against 3D-PTV for relatively high-amplitude sinusoidal inputs and presented in Figure 8. Once again, the 3D-PTV results correlate well with both sensors and essentially offer a lower noise level than the LVDT. Power spectral densities of the 3D-PTV measurements, following a low-level hammer impact producing subtle (imperceptible) motion, are provided in Figure 8. Initial observations hint that subtle motions are not well captured with 3D-PTV, likely because of the image splitter setup reducing the resolution from 1024 × 1024 to 512 × 512 pixels, as well as the out-of-plane accuracy being lower than that of the in-plane [62]. Similar to the 2D case, the PTV signals contain harmonic peaks in the frequency domain.

### 3.3. Magnified Tracking

In order to validate the proposed MT framework, a video captured via the stereoscopic splitter setup was magnified within frequency bands determined from a single triaxial accelerometer. These frequency bands are defined around natural frequencies of the structure, i.e., around peaks determined from the power spectral density estimate. The original video contains the structural response induced by a low-amplitude excitation via an impact hammer, in which displacements due to this excitation are imperceptible to the naked eye and lie below the tracking capability of 3D-PTV. The PBMM algorithm was then employed in order to amplify motion around the defined frequencies and thus reveal their operational deflection shapes, which approximate the structural mode shapes, as perceptible motion in the video. The chosen frequency band ranges were 7–9 Hz, 22–23 Hz, 26.5–28.5 Hz, and 33–34 Hz, respectively. These bands were manually adjusted to be as broad as necessary for yielding the full magnified motion [56]. Magnification factors that result in high-amplitude perceptible motion in videos without introducing excessive artifacts were determined manually. This factor, previously defined as α in Equation (7), was determined to be 50, 150, 100, and 250 for each mode, respectively. The magnified videos were then fed into the 3D-PTV algorithm to track the motion of markers and extract the aforementioned modal information. The original and motion magnified videos are provided in the online version of this manuscript as Supplementary Material.

**Figure 9:**
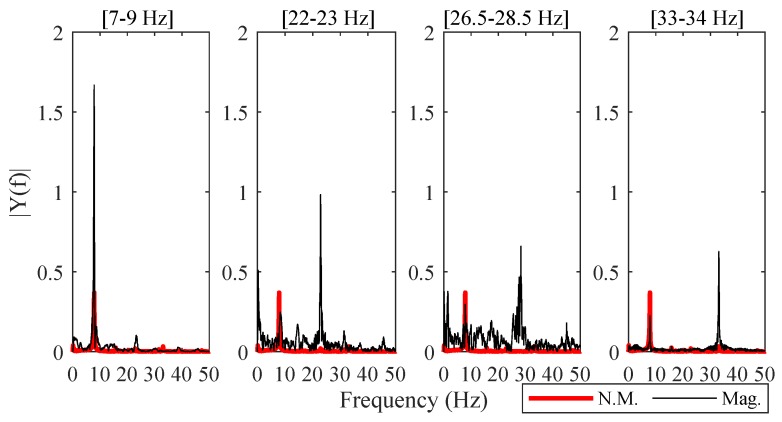
Fourier spectra comparison of tracked motion obtained through non-magnified (N.M.) and magnified videos.

The effect of PBMM in the frequency domain is visualized in Figure 9. Each sub-figure, corresponding to videos magnified between the above-mentioned frequency bands, comprises the Fourier spectra of tracked motion from a selected marker. It is evident that the frequency range at which the videos were magnified experience a significant boost. As with other techniques, the identified resonant peaks via stabilization diagrams for the 3D-MT framework are listed in Table 2.

**Figure 10:**
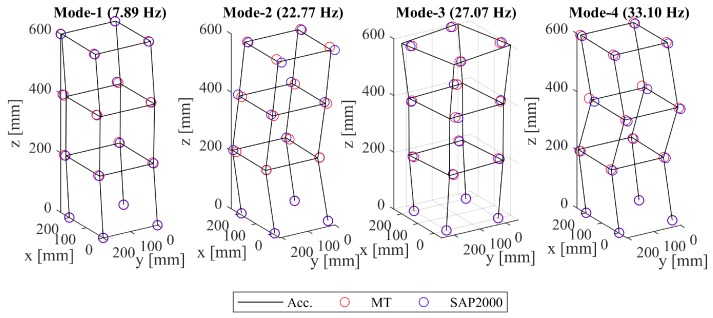
Comparison between the normalized mode shapes obtained via accelerometers and SAP2000, and the operational deflection shapes via 3D-MT using a “coarse” grid (scaled for better visualization).

A 3D modal analysis was carried out, in which: (i) operational deflection shapes from 3D-MT were compared against modal shapes obtained from accelerometer measurements and SAP2000 simulations at each storey; and (ii) operational deflection shapes from 3D-MT were compared against the mode shapes obtained via SAP2000 at a higher spatial density, including two additional nodes per column. As previously mentioned (see Figure 5), the conventional accelerometer-based modal analysis was realized via a reference-based technique because of the limited channel availability. Reference-based stochastic subspace odentification (SSI) [63] was employed to this end. The operational deflection shapes obtained via 3D-MT, the mode shapes computed via accelerometer measurements, as well as the SAP2000 structural model are presented in Figure 10. 3D-MT delivers a precise replication of mode shapes compared to the conventional experimental techniques, with the added advantage of alleviating the need for a reference-based measurement setup because of the absence of “channel” constraints. Furthermore, the torsional mode shape was recovered, which was obviously non-existent via the 2D measurement setup. The cross-MAC values are close to one, as illustrated in Table 3, thereby demonstrating the precision of the proposed framework when compared against conventional accelerometer-based sensing.

**Figure 11:**
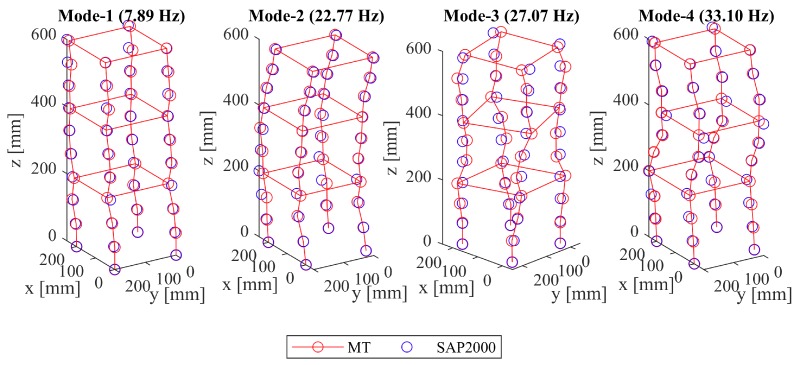
Comparison between the normalized mode shapes obtained via SAP2000 and the operational deflection shapes via 3D-MT using a “fine” grid (scaled for better visualization).

As previously pointed out for the 2D setup, a main advantage of adoption of computer vision techniques for dynamic measurement applications lies in its potential to obtain displacement information of high spatial density. For this purpose, an additional analysis was carried out using the 3D-MT data enhanced with intermediate markers placed at the columns. Since no information from the accelerometers exists at these points, the operational deflection shapes are compared only against the SAP2000 simulation results and are presented in Figure 11. These shapes, which were yet again obtained without the limitations imposed by the number of available channels in conventional techniques, agree well with the mode shapes obtained via the SAP2000 simulation results. This indicates that the proposed method can be utilized to obtain modal information at locations where no conventional sensors may be placed, thereby increasing the potential of damage detection and localization. The cross-MAC values of the proposed framework, when compared with the SAP2000 results, given in Table 3, deliver values close to unity, further confirming its feasibility as a tool for high spatial density modal information.

## 4. Conclusions and Outlook

A novel framework for obtaining high spatial density dynamic displacements as well as modal information both in 2D and 3D was proposed and validated against conventional sensing technologies. This framework relies on the synergistic use of two computer-vision techniques for system identification, namely PBMM, to amplify imperceptible motion in recorded videos, and PTV, to track markers placed on the structure. A three-storey shear frame was tested for this purpose, simultaneously measured by means of conventional (reference) sensors. Perceptible displacements obtained through PTV both in 2D and 3D were validated against LVDT and laser distometers. Validation in the frequency domain was achieved by comparison of the power spectral densities of the proposed methods and the reference measurement system of the accelerometers.

The conducted modal analysis further revealed the identification potential of the proposed framework, since high spatial density information was acquired without the need for reference-based system identification techniques. When videos are processed via PBMM, the resulting magnified motion offers the operational deflection shape of the structure within the amplified frequency band, which in turn approximates the corresponding structural mode shapes.

A possible limitation for outdoor implementations is related to the fact that PTV requires a certain contrast between the background and the object, implying that a well separated background needs to be provided. Moreover, the presented image splitter setup needs to be modified, i.e., a bigger splitter and larger mirrors ought to be used, to capture the response of large scale structures from a sufficient distance. The latter issue can also be addressed through the use of multiple cameras.

## Figures and Tables

**Table 1:** List of experiments for different excitations and measurement techniques compared with the reference technique.

Experiment No	Excitation	Dimension	Measurement Technique	Reference Technique
1	Northridge	2D	2D-PTV	Linear variable differential transformer (LVDT)
2	Hammer	2D	2D-PTV	Laser
3	Hammer	2D	2D-PTV	Accelerometers
4	Sinusoidal	3D	3D-PTV	Laser & LVDT
5	Hammer	3D	3D-PTV	Accelerometers
6	Hammer	3D	3D-MT	Accelerometers

**Table 2:** Identified natural frequencies via different sensors and methodologies.

	Identified Frequencies [Hz]			
**Acc.**	7.89	22.77	27.07	33.10
**2D-PTV**	7.90	22.90	-	33.31
**3D Magnified tracking (MT)**	7.86	22.90	27.15	33.01

**Table 3:** Cross-MAC values of the mode shapes and operational deflection shapes obtained via 2D-PTV and 3D-MT, respectively, versus the mode shapes from the accelerations and SAP2000 modal analysis.

	2D-PTV			3D-MT			
	Mode-1	Mode-2	Mode-3	Mode-1	Mode-2	Mode-3	Mode-4
**Accelerometer**	0.9925	0.9855	0.9850	0.9641	0.9229	0.9535	0.9986
**SAP2000**	0.9871	0.9883	0.9811	0.9388	0.9329	0.8862	0.9290

## Supplementary Materials

The following are available online at https://www.mdpi.com/1424-8220/19/5/1229/s1, Video S1: Original input video and resultant videos following motion magnification. Frame rates are reduced for a better visualization of the operational deflection shapes, which are denoted below each individual video.

## Abbreviations

The following abbreviations are used in this manuscript:

DIC	Digital Image Correlation
MT	Magnified Tracking
PTV	Particle Tracking Velocimetry
PBMM	Phase-Based Motion Magnification
SSI	Stochastic Subspace Identification
ERA	Eigensystem Realization Algorithm
